# Software for improved field surveys of nesting marine turtles

**DOI:** 10.1038/s41598-017-11245-6

**Published:** 2017-09-07

**Authors:** R. Anastácio, J. M. Gonzalez, K. Slater, M. J. Pereira

**Affiliations:** 1 Departamento de Biologia e CESAM, Universidade de Aveiro, 3810-193 Aveiro, Portugal; 2 Centro Ecológico Akumal, Akumal, Tulum, Quintana Roo, CP 77780 Mexico; 3 Operation Wallacea, Wallace House, Old Bolingbroke, Lincolnshire, PE23 4EX England; 4 AFPR – Oceans, Aveiro, Portugal

## Abstract

Field data are still recorded on paper in many worldwide beach surveys of nesting marine turtles. The data must be subsequently transferred into an electronic database, and this can introduce errors in the dataset. To minimize such errors, the “Turtles” software was developed and piloted to record field data by one software user accompanying one *Tortuguero* in Akumal beaches, Quintana Roo, Mexico, from June 1^st^ to July 31^st^ during the night patrols. Comparisons were made between exported data from the software with the paper forms entered into a database (henceforth traditional). Preliminary assessment indicated that the software user tended to record a greater amount of metrics (*i.e*., an average of 18.3 fields ± 5.4 sd vs. 8.6 fields ± 2.1 sd recorded by the traditional method). The traditional method introduce three types of “errors” into a dataset: missing values in relevant fields (40.1%), different answers for the same value (9.8%), and inconsistent data (0.9%). Only 5.8% of these (missing values) were found with the software methodology. Although only tested by a single user, the software may suggest increased efficacy and warrants further examination to accurately assess the merit of replacing traditional methods of data recording for beach monitoring programmes.

## Introduction

Considering the need for global commitment and engagement, science and technology currently play an important and a probably decisive role. Conservation means eternal vigilance regarding an ecosystem^1^. Ecologists have multiple strategies to implement conservation; some of them are exciting, new and technological. Monitoring ecosystems has become the centre of attention due to pressures that affect their equilibrium, such as climate change, disturbances in the mass-energy flow from producers to top consumers^2^, and competition for resources between wild species and humans^3^. Monitoring is also labour intensive due to the number of variables that must be measured and the speed at which analytical delivery must act^4, 5^. Different high-performance technologies that improve daily life have been developed for citizen use during this century, for example, smartphones, apps, laptops, tablets, and drones. These technologies can be or are associated with wildlife protection based on a real-time survey, for example, of rhinoceros (www.cisco.com/c/m/en_us/never-better/csr-1.html) or elephants (www.savetheelephants.org/). These technologies can also be used by investigators to facilitate field work^6^ or to help advance current knowledge (*e.g*., about elephant communication^7^).

Marine turtles have been the target of numerous conservation projects that require monitoring and data collection to understand population dynamics and trends^8–10^. This in turn requires a large amount of data and several years of monitoring, particularly for long-lived species such as marine turtles^9^ (see refs 11–14 for examples of monitoring studies). The monitoring of marine turtles particularly poses considerable challenges due to their behaviour during their early years (oceanic stage) or in the juvenile and adult phases because the turtles travel between breeding and feeding areas (transoceanic migration)^12, 15, 16^. Additionally, adult males do not visit beaches during the nesting season^17, 18^, and distinguishing between male and female hatchlings requires invasive techniques (*e.g*., a histological analysis)^17^. Such complexities have required the development of a range of monitoring strategies, such as capture-mark-recapture using metal or plastic tags^19^, or more advanced technology, such as PIT tags^19^, satellite and molecular tracking^16^, and molecular techniques^20^. Still, considerable questions remain unanswered^10^.

Many conservation projects monitor nesting beaches where a group of females come every year to nest. Monitoring the nesting females, their nests and hatchlings (the nesting beach surveys) is not sufficient for characterizing a population, but such surveys provide important indicators of the population status and assist in the development of local conservation management measures for those individuals at the beach or in the surrounding area (*i.e*., in the neritic habitats and corridors). Standardized procedures are important because many projects provide basic data for conservation measures. The State of The World’s Sea Turtles (SWOT)^9^ provides information on global standards for data reporting. SWOT intends to build an improved data collection by relying on a global network of data providers for all aspects of sea turtle biogeography^9^.

The current trend is the assembly of all information in a single database, especially for globally distributed migrating species^21^ such as marine turtles. The study of multi-scale Regional Management Units (RMUs) by Wallace *et al*.^22^ exemplifies both the asymmetry in data worldwide, which are biased towards areas in which monitoring and reporting are high, and issues with the data quality provided. Wallace *et al*.^22^ conclude that the efficacy of applications using RMUs is dependent on the accuracy and quality of the data contained in the files, including the difference between the true absence of a species and an apparent absence due to a lack of monitoring or reporting. Similarly, other studies have shown that the scientific effort is skewed, and some programmes lack scientific guidance^8, 16, 20, 23, 24^.

It is necessary to develop a global database for marine turtles in order to understand their distribution and population trajectories. Information for the Western Indian Ocean (WIO), for example, is lacking^25^. The *IUCN East African Regional Office (EARO)* and the *IUCN/SSC Marine Turtle Specialist Group* ^26^ have previously emphasized a need to improve monitoring efforts in this region to achieve more and better reporting.

Following the recommendation made regarding the WIO^25^, and as a consequence of previous work^27^, a software tool was developed to help teams in remote areas gather and report information to entities such as SWOT. A software tool used to record field information that uses adequate methodology for a certain nesting season and follows SWOT^9^, Eckert *et al*.^17^, Sims *et al*.^15^, and WIDECAST^28^ recommendations may provide an enormous benefit for conservation projects compared to existing monitoring that employs paper.

The premise was that, in order to enhance data quality, it is important not only to choose adequate protocols and methods^9, 15, 17, 28^ but also for field ecologists to use field technology, such as software prepared for a specific purpose, to collect variables.

A new way of executing field ecology has arisen due to the development and use of software applications for ecological monitoring. Some projects, such as iNaturalist (www.inaturalist.org/), iBats (www.bats.org.uk/pages/ibatsprogram.html), and iBird (ibird.com/), can serve as examples. Actually, the window of opportunity is open for ecologists to create and test monitoring software for many species. The Bruna Lab (brunalab.org/apps/) has a list of free applications for ecologists. More importantly, this indicates that traditional methodologies will be replaced by digital tools in field ecology for the monitoring of many species.

Sims *et al*.^15^ emphasize that counting errors by an observer and environmental stochasticity have an impact on trend determination for marine turtles. Software that standardizes the way a field ecologist saves data also enables the possibility of comparing information over time, since it helps reduce errors due to manual data re-entry^6^. The same can be said for the use of integrated tools; instead of recording GPS coordinates manually on a paper form, a field ecologist can use a GPS app to save that information and avoid errors.

To examine the previous assumptions, a software package named “Turtles” was developed and piloted in the field as a method for data recording for subsequent statistical analysis. Its performance was trialled in conjunction with the traditional methodology (i.e., paper forms and entered into a database). The goal is to replace traditional data recording methodology with dedicated software, such as an app for a tablet or a smartphone, that provide significant benefits for monitoring of marine turtles nesting.

## Results and Discussion

Two months of field work from June 1^st^, 2016, to July 31^st^, 2016, were recorded and analysed comparatively. During this period, *Centro Ecologico de Akumal* (CEA, see Methods section) kept their traditional methodology of data collection on paper forms, while the software-user used the developed software for the same task. Then, the data collected by the 3 main *Tortugueros* (a specific designation given to field monitors that work only with turtles, which in Mexican are called “tortugas”) from CEA was used as benchmark to assess the performance, effectiveness and benefits of the software tool for field work.

Table 1 shows that the software database contains a total of 171 records (the effort of one person in the field). The paper forms database contains 561 records (the effort of three people in the field). These records were scrutinized in terms of “missing values” and “errors” amounts and percentages.

**Table 1:** Analysis of missing values and “errors” of the paper forms and software databases.

**Description**	Paper forms database		Software database	
	**Records**	**Percentage**	**Records**	**Percentage**
Total amount of collected data (from 01/06/2016 to 31/07/2016)	561	100.0%	171	100.0%
Field effort	3 People		1 Person	
Total amount of collected data per person	187		171	
1. Missing value for relevant fields:				
1.1. GPS location of the Nest	176	31.4%	10	5.8%
1.2. Time (hh:mm) of the record	53	9.4%	0	0.0%
1.3. User name	5	0.9%	0	0.0%
1.4. Nest ID	1	0.2%	0	0.0%
**Total**	**225**	**40.1%**	**0**	**5.8%**
2. Error “different answers for the same value”:				
2.1. Different texts for the same user	49	8.7%	0	0.0%
2.2. Different texts for the same turtle false crawl reason	6	1.1%	0	0.0%
**Total**	**55**	**9.8%**	**0**	**0.0%**
3. Error derived from “inconsistent data”:				
3.1. Mismatch between total number of eggs and the sum of the partials	2	0.4%	0	0.0%
3.2. GPS data out of the range of that region	3	0.5%	0	0.0%
**Total**	**5**	**0.9%**	**0**	**0.0%**
**Total number of records with errors**	**262**	**46.7%**	**10**	**5.8%**
Total number of records without errors	299	53.3%	161	94.2%
**Total number of records without errors per person**	**99.7**		161.0	

The total number of records with errors is not a sum of the values of the column above but rather the total number of records that contain that error. For example, if a single record contained multiple errors, they were counted as a single error.

The percentage of missing fields in the software database is 5.8%, whereas the number of missing fields in the paper forms database is higher, at 40.1% (see the “Methods” section for the definition of “record” and “field” in the databases). For example, 9.4% of the paper forms database did not contain the hour (when the record was made) (Table 1), and one record had a missing nest tag. The software always showed a list of nest tags (the tag is introduced at the moment the nest is identified during the field work, though the software generates a redundant automatic tag for it, enabling the user to check the nest codes and avoiding the duplication of nest tags, which is an advantage). The missing fields in the software database related to the GPS location were due to the fact that the coordinates were introduced manually. This issue will be addressed in a software upgrade to reinforce and automate the capture of these data. Although there is a significant effort to revise the software source code, the use of software and technological tools provides the benefit of continuous improvement of the process, with a minor impact on the field effort performed by the *Tortugueros*. Therefore, when the software database is compared with the information collected on paper forms, the absence of data on relevant fields is higher in the traditional paper approach. Thus, the software performed better and added a benefit.

No errors derived from “inconsistent data” were found in the software database. On the other hand, 0.9% of data inconsistencies were found in the paper forms records. Hence, the total number of paper forms records that were error-free was 299. Although the number of records per person was higher for *Tortugueros* (16 more field form records than in the software database), the software user generated fewer misses (5.8% versus 46.7% on paper forms) and 0% errors (errors type two and three in Table 1). This translates into a greater number of valid records per person (161 software records versus 99.7 records by traditional method), which is a similar efficiency of absolute data recorded in the field. However, the data recorded by the software user was more reliable and of higher quality.

The errors found frequently originated in the traditional methodology (as expected from previous experience), which is prone to errors due to higher process exposure to potential sources of error than in the software approach. These derive from 1) different field content that represents the same information, *i.e*., the same result was given different designations (for example, the names of *Tortugueros* were given different designations, or empty fields were identified by different symbols, which causes problems and requires time-consuming work to prepare the file for statistical analysis); 2) missing information requiring another result; or 3) typing errors that implied an unrealistic data range, the wrong coordinates, or duplicated nest tags. These “errors” were negligible in the software database, since several mechanisms were introduced in the application to avoid them. The mechanisms of the software were multiple choice fields (weather conditions in several variables, species ID), mandatory fields (the user can only proceed if certain variables are filled in), scrolling information (for example, a list of the introduced nest tags is presented to the user when it is necessary to introduce new coordinates for the nest, when it is moved, and when the nest is excavated, after eggs hatched), and auto-filled information fields (date, time).

Time is an important variable when comparing the performance of the traditional methodology with the software. The standardization of the paper forms database (correction of “errors”, when possible) required three working days. However, when the software database was ready for analysis, the paper forms database had to be prepared by entering information on paper forms into a computer, which required several weeks.

For the results in Tables 2 and 3, the analysis of the two databases was divided into four categories: “turtles seen or not seen (false crawls)”, “turtles seen”, “nests seen” and “nests moved”. The software user filled in 18.3 fields ± 5.4 sd on average in a single record with data of marine turtles, versus 8.6 fields ± 2.1 sd on average for *Tortugueros* (Table 2). This is because the software allows more data to be recorded if the user so desires (the average number of fields recorded by the software user was higher or similar in all four categories in Table 2). For example, the software user always recorded weather variables (which are not indicated in Table 2), while *Tortugueros* rarely collected weather information. This reveals the power of the digital tool *versus* paper forms.

**Table 2:** Average number (±standard deviation sd) of fields filled in *per* record according to the category of analysis.

	**Category of analysis**			
	Turtles seen or not seen (false crawls)	Turtles seen	Nests seen	Nests moved
Average number of fields (±sd) filled per record in the paper forms database	8.6 ± 2.1	10.5 ± 1.1	13.9 ± 7.2	14.9 ± 6.0
Total number of fields in the paper forms	15	15	15	19
Average number of fields (±sd) filled per record in the software database	18.3 ± 5.4	22.9 ± 4.7	25.7 ± 5.1	14.0 ± 0.8
Total number of fields in the software Turtles	23	57	49	22
fields / variables considered for this analysis (examples)	Species id; Tag number; CCL (curved carapace length); CCW (curved carapace width); Track width; Activity of the turtle.	Species id; Tag; CCL (curved carapace length); CCW (curved carapace width); Track width; Activity of the turtle	Nest tag; GPS coordinates; Temperature inside the nest;Temperature of the sand; Nest depth	GPS of the new location; Nest tag; Number of eggs; Number of broken eggs

Notice that not every field must be filled in every record for each category. The software was created taking into account the possibilities of every monitoring project; for example, it has fields for passive integrated transponder (PIT) tags information, and not every monitoring project uses PIT tags. In the Akumal Project, measurements of total tail length (TTL) and post-cloacal tail length (PTL) are not taken, though the software has an entire window to insert that information. This is the reason why the total number of fields to fill in is always greater than the averages presented.

**Table 3:** Number and averages (±standard deviation sd) of records of the entire databases, collected in two months, by the *Tortugueros* and the software user.

Categories of analysis	Number of records of turtles^1^	Number of records of turtles seen	Number of records of nests	Number of records of nests moved
Averages ± sd of CEA Top 3 *Tortugueros*	65.2 ± 20.5	33.3 ± 3.7	36.7 ± 9.7	4.0 ± 2.0
Averages ± sd of software user	74.5 ± 13.5	34.0 ± 5.0	25.0 ± 2.0	2.5 ± 1.5

^1^Includes false crawls.

Upon analysing the average number of records per category (four categories considered) of the entire databases (Table 3), it is possible to find that the software user made more records of “turtle activities” and of “turtles seen” when compared with the top three *Tortuguero*s averages (74.5 ± 13.5 records *versus* 65.2 ± 20.5 records and 34.0 ± 5.0 records *versus* 33.3 ± 3.7 records, respectively). This shows that the software does not disturb the work of the user or even diminish its performance. The average of the two categories involving nest variables was lower (Table 3) for the software user compared to the *Tortugueros*. The reason that fewer records were shown for nest variables has more to do with the field division of tasks than with the capacity of the software user to record them, as well as with the hatching dates of the eggs (after the final stage of the pilot study).

The functionality of the software on a tablet on the beach at night proved to be very reliable. Both methods are practical; both are functional for field data recording. However, Table 4 shows the specific differences between them.

**Table 4:** Characteristics of the traditional methodology compared to the software.

Traditional methodology – paper forms	“Turtles” software
After the data are recorded on paper forms, they must be entered in an Excel file.	After data about a turtle, a nest or a crawl have been recorded, a table containing that information is automatically provided.
Does not introduce date and hour automatically.	Introduces date and hour automatically.
The user must write the same thing repeatedly, even if it is the same information.	Has selectable, predefined answers, and new entries can be added.
The user must write in each paper form for every variable.	Enables the collection of several weather datasets only once per night (at the beginning of the recording), but more information can be added at different times (changes, for example). If no changes are observed, the software automatically replicates the weather information entered in the beginning in each record.
Does not have a mechanism to avoid the duplication of nest tags; the last record must be consulted before going to the field.	Avoids the duplication of nest tags.
A red light is necessary for recording on paper.	No red light is necessary - the light of the tablet/smartphone is sufficient.
It is not dependent on a battery source, but a pencil or pen (that the user sometimes loses) is necessary.	Needs a battery (prior charging required) or power bank.
If all the paper forms are used, more are necessary to continue recording data. *Tortugueros* use their smartphone or a notebook in these situations.	No need for backup. Works until charge runs out.
Cannot be used if it rains (the paper gets wet).	Can be used in light rain.
Difficult to use in windy conditions.	Not difficult to use in windy conditions.

The results suggest that the software has several advantages compared to the traditional methodology. First and foremost, it avoids the need for data entry on a computer after it has been collected on paper. The dataset provided by the software is immediately available for analysis by a statistical tool because data consolidation is not required.

Another aspect in favour of replacing paper forms with the Turtle software is that the *Tortugueros* use a smartphone on the beach to communicate with each other and even to record data after all of their paper forms have been exhausted. Thus, *Tortugueros* were at ease with the software and were satisfied with the idea that they did not have to type the information on the office computer. It is important to emphasize that the user can manipulate the database produced by the software at any time, even on the beach.

## Methods

### Akumal Project

The Sea Turtle Protection Program of the Akumal beaches was created in 1993 (www.ceakumal.org), and its development is carried on by the CEA. Although this protection programme focuses on nesting and feeding grounds^29^, nesting site preferences of female green sea turtles (*Chelonia mydas)* and loggerhead sea turtles (*Caretta caretta*) were the focus of this study.

CEA is the local Non-Governmental Organization with permission for managing the natural resources of the newly created marine protected area and beaches. As of March 7^th^, 2016, an agreement that establishes “Bahía de Akumal” as “Área de Proteccion de Especies Marinas” in the Tulum Municipality of Quintana Roo State^29^ was decreed by the Mexican Government. This refuge area was created for the protection of the following species of sea turtles: *Chelonia mydas*, *Caretta caretta*, *Eretmochelys imbricata*; corals: *Acropora palmata*, *Acropora cervicornis*, *Plexaura homomalla, Plexaura dichotoma*; mangrove and dune species: *Laguncularia racemosa, Rhizophora mangle, Conocarpus erectus;* and seagrass species: *Thalassia testudinum*, *Syringodium filiforme, Halodule wrightii* ^29^.

The research objectives of the nesting part of this project were 1) to determine the physical characteristics of the beach female sea turtles preferred for nesting at Akumal; 2) to determine how the physical characteristics of the nest relates to the ambient temperature inside the nest; and 3) to use these results to predict the sex ratios of the hatchlings in Akumal^30^. The results can then be used to determine if adaptive management of the nests should be implemented in the future^31^. This work was carried out in collaboration with CEA and Operation Wallacea under the *Sea Turtle Protection Program* based in Akumal, Mexico.

### Turtle Surveys

**Figure 1:**
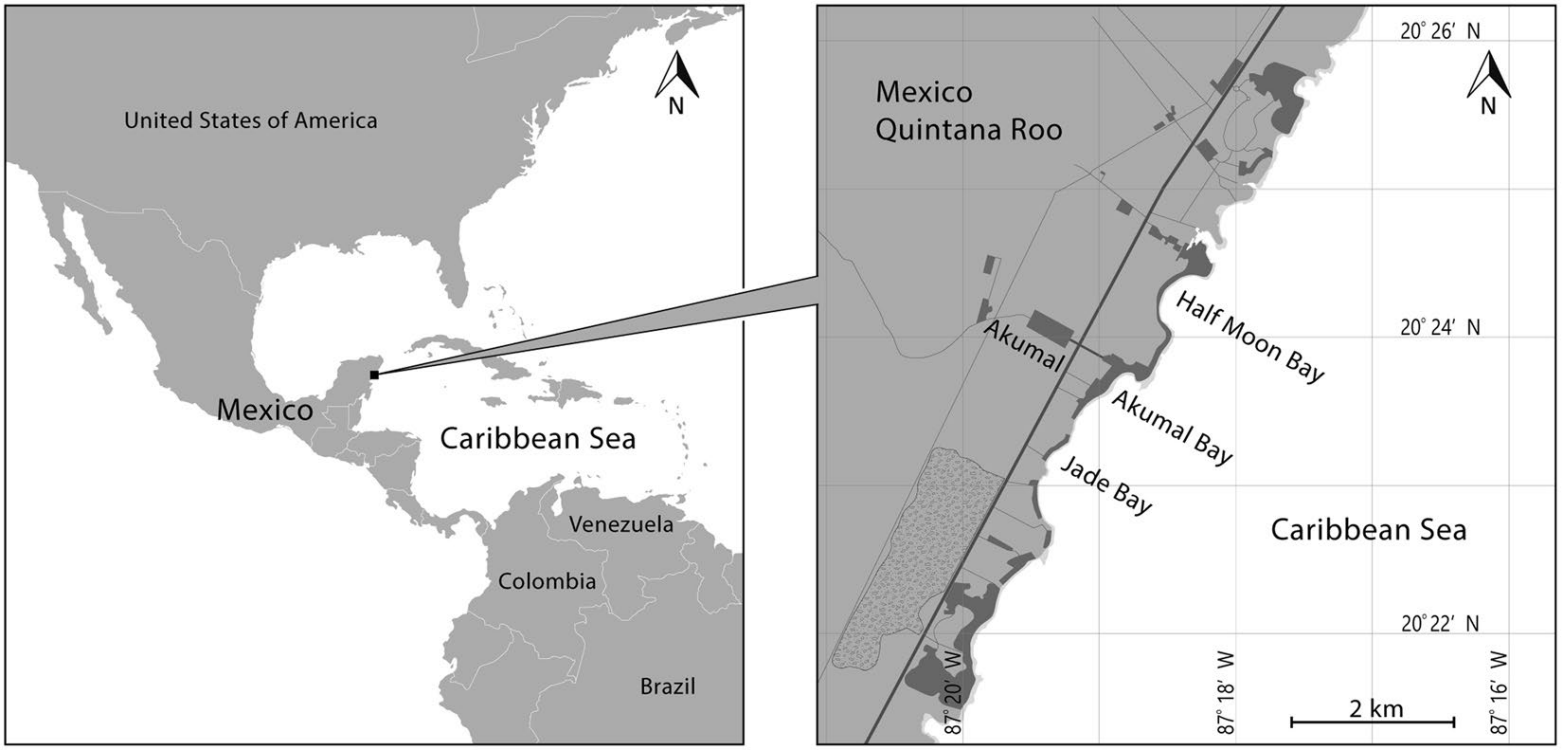
Location of Akumal and the beaches. The field work occurred primarily on Half Moon Bay beach, which was divided into 7 sectors, and Jade Bay beach, which was divided into 5 sectors. Left image designed by Freepik (http://www.freepik.com) and modified with Adobe Illustrator CC2017. Right image designed with Adobe Illustrator CC2017 from Landsat image; Landsat imagery courtesy of NASA Goddard Space Flight Center and U.S. Geological Survey.

The nesting season in Akumal occurs from May to November or December^30–32^. During those months, the *Tortugueros* patrol the beaches every night from 9 p.m. until 4 a.m. or longer, depending on the nesting activity. They also check the nests during the day at 6 a.m., when the clutches start to hatch. Each patrol is composed of 1 or 2 *Tortugueros* per beach (Fig. 1) at Half Moon Bay, Akumal Bay, Jade Bay, and South Akumal. Each beach is divided into sectors marked with a divider every 100 metres.

The number of *Tortugueros* varied during the season, but CEA had 4 permanent *Tortugueros* and hired one more permanent *Tortuguero* in July. In June and July (2016), 7 volunteers helped during the night patrols, so the field effort encompassed 4–12 people distributed on the four beaches. During each night patrol, the *Tortugueros* patrolled the beaches and specifically identified nesting attempts, nesting turtles and nests. The variables collected during the 2016 nesting season (the same variables reported in CEA-OpWall report^30^) were the weather conditions, the tide level, moon phase, cloud cover and artificial light for each night. For each nesting female, *Tortugueros* recorded the species of turtle, the time and date when the female began to lay eggs, the nest number, the curved carapace length (CCL) and width (CCW), the presence of neophytes on the carapace, and the number of the tag located on the front flipper, where applicable^30^. The *Tortugueros* collected the following nest variables: the nest depth (with a hard measuring rule) and the nest temperature (with a pen thermometer, 0.1 °C) at 2 cm into the sand and at the bottom of the nest (usually at 40 cm deep using a hard tape measure that was inserted inside the nest)^30^. The *Tortugueros* also collected variables from sites in the zones where nest densities were low and at sites rejected for nesting. These variables were primarily the sand temperature at 2 cm depth, obstacles in the potential nest zone (*i.e*., none, natural, or man-made), obstacles in the tidal zone, artificial light, human disturbance (*i.e*., none, man-made obstacle, human obstacle, human voices, human presence)^30^. The CCL measured was the CCL notch to tip (n-t) according to Bolton’s^17^ methodology, *i.e*., the length “from the anterior point at midline (nuchal scute) to the posterior tip of the supracaudals”^17^ of the carapace. GPS UTM coordinates were taken with a Garmin e Trex® 10, written on paper forms and entered into the software manually.

The turtles were tagged according to the Eckert and Beggs^33^ methodology. The project used Monel tags^33^ that were generally applied to the front left flipper after checking the flipper and the paddles for tags and scars. Turtles with obvious fibropapilloma (FP) disease were documented but not tagged^19^.

### “Turtles” Software

The “Turtles” Software was developed as a tool to monitor the activity of nesting females from several species, the leatherback (*Dermochelys coriacea*), the hawksbill (*Eretmochelys imbricata*), the green (*Chelonia mydas*), the loggerhead (*Caretta caretta*), the Kemp’s ridley (*Lepidochelys kempii*), and the olive ridley (*Lepidochelys olivacea*) turtles, following recommendations by the “Research and Management Techniques for the Conservation of Sea Turtles”^17^ and others^8, 9, 27, 28^. The software, designed and built with the mission of simplifying the data recording task on field and improving the quality of the data collected, was used in this scenario as a “proof of concept” tool.

Its functionality was tested with simulations by navigating it (testing buttons and paths) and fulfilling every variable. Databases generated during the simulations were carefully analysed to detect malfunctions, errors, and mismatches. The development process and tests took six months. After the development process, the software was used and tested in Akumal during two months of field work. The software was built to satisfy the need to enter as much data as possible, following the methods of Eckert *et al*.^17^. However, each project has only a part of those variables, and the Turtles Software can be adapted to those specifications. For example, the software window about the turtle species gives the possibility of recording information on Leatherbacks’; however, it was not necessary to record information about that species. In the future, a version of the software can be provided for each project, according to the specifications and needs of the users.

A version to record information only on tracks or only on nests is possible; the version used is the most complete, with all the possible variables so far. The software can record a total of 104 variables, concerning weather conditions, abiotic variables (such as the sand temperature in the nest, sand humidity, sand temperatures), the GPS coordinates of the tracks and of the nests, all the variables concerning nesting turtles (CCL, CCW, track width, post-cloacal tail length PTL, total cloacal tail length TTL, head length, new tags, old tags, health conditions, among others), nest (depth, number of eggs per category, number of nests in its proximity, tag, obstacles near the nest, predators) and track variables (type, width, causes of false crawl).

The software was developed using Microsoft Visual Studio Community 2015, in Visual Basic language. The source code was compiled and installed on two 7-inch tablets running Windows 10 Operating System, which were used during the field work. The software records the data in a local Microsoft Access file. Its interface consists of several windows with relevant monitoring variables.

**Figure 2:**
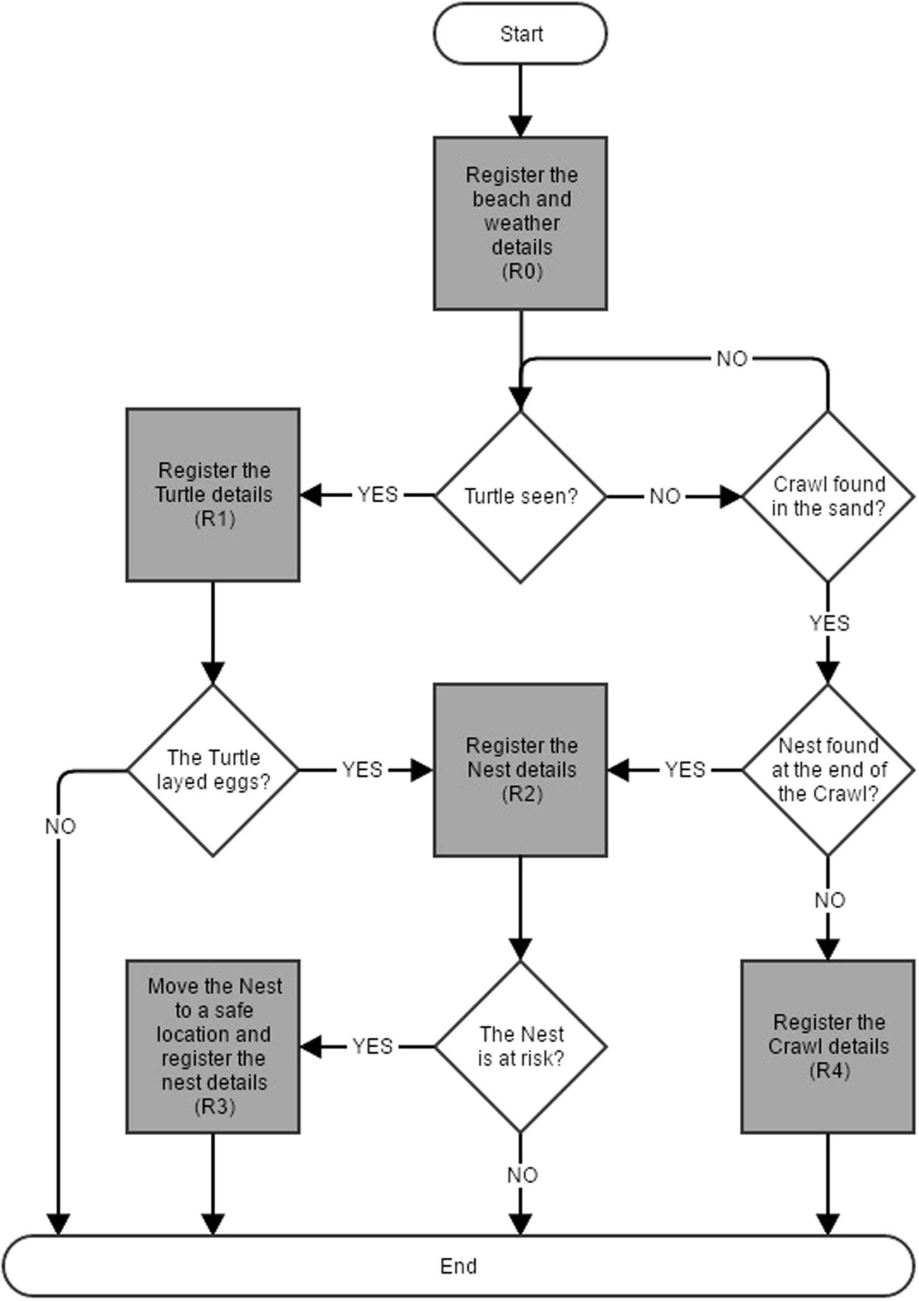
Workflow for the field work of a nesting turtle project. The software was built with the same logic.

The tool guides the user according to the work flow of the field teams, as shown in Fig. 2. It is possible to make several records in sequence after the R0 box that comprises all windows concerning weather and disturbance factors. The R1, R2, R3 and R4 fields allow several data types to be recorded; for example, if an investigator has a turtle laying eggs, and if those eggs have to be moved, the software will allow that to be recorded in sequence in the R1, R2 and R3 fields. However, if the investigator chooses to save only information about the nest or to enter that information before entering information for a turtle, he can use that approach. More importantly, at the end of each box of variables (R1, R2, R3 or R4), it is mandatory to save information before going to another box.

**Figure 3:**
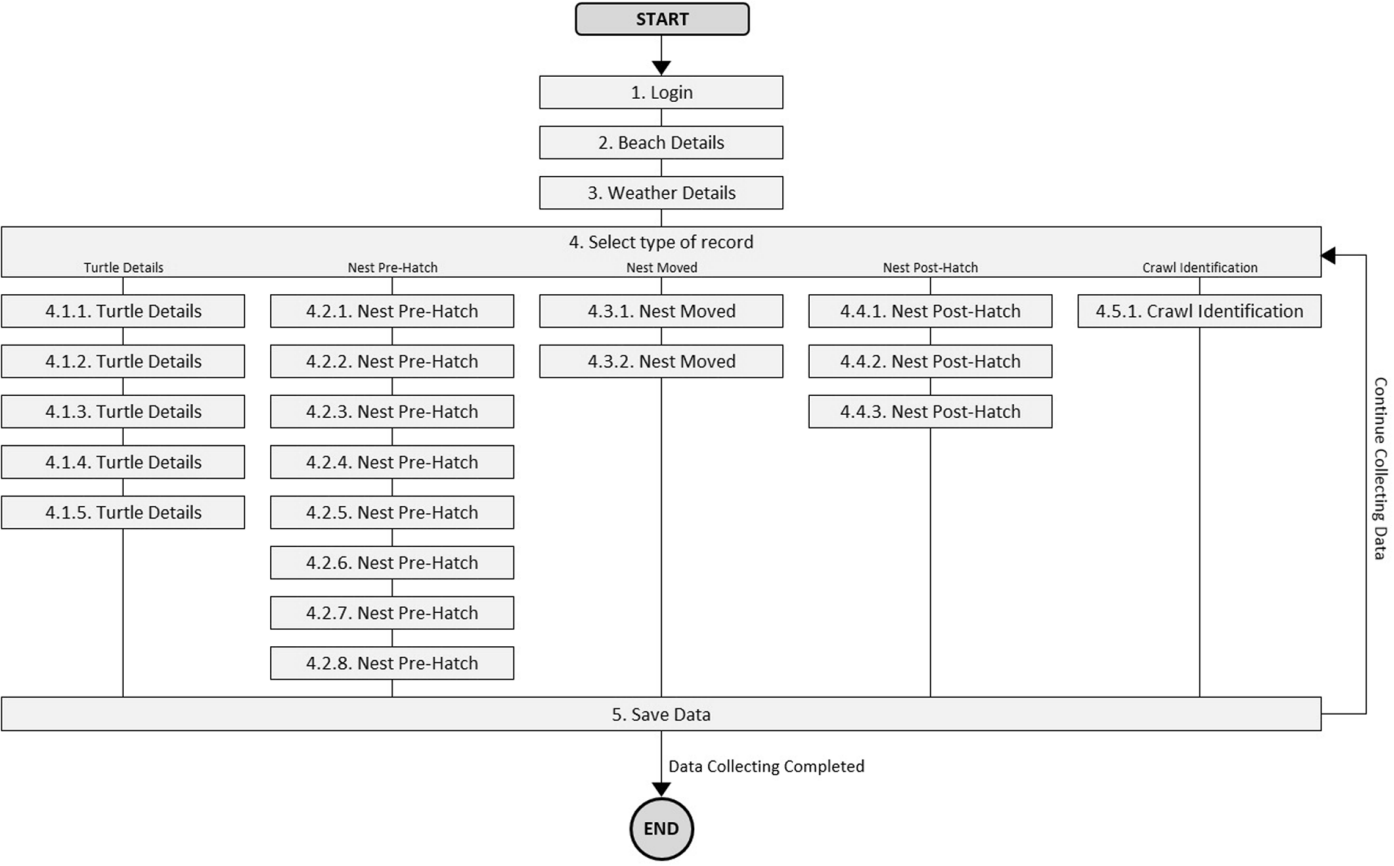
Software structure of windows offered to the user.

The flow of windows shown to the software user is demonstrated in Fig. 3, which is a complement of Fig. 2. When started the software, the user will find the “1. Login” window to type in identification and password code. After authentication, the user will find the “2. Beach Details” and “3. Weather Details” windows to fill in the generic information of the place. Then, in window 4, the user must select the type of record according to the situation on the field. As shown in Fig. 3, the selection made in window 4 will direct the user to the corresponding set of windows. Between windows 2 “Beach Details” and 5 “Save data”, the user can move forward and backwards along the multiple windows. For multiple records (*i.e*., turtle seen and nest found), the software allows the user to “Continue Collecting Data” after completing and saving a record.

**Figure 4:**
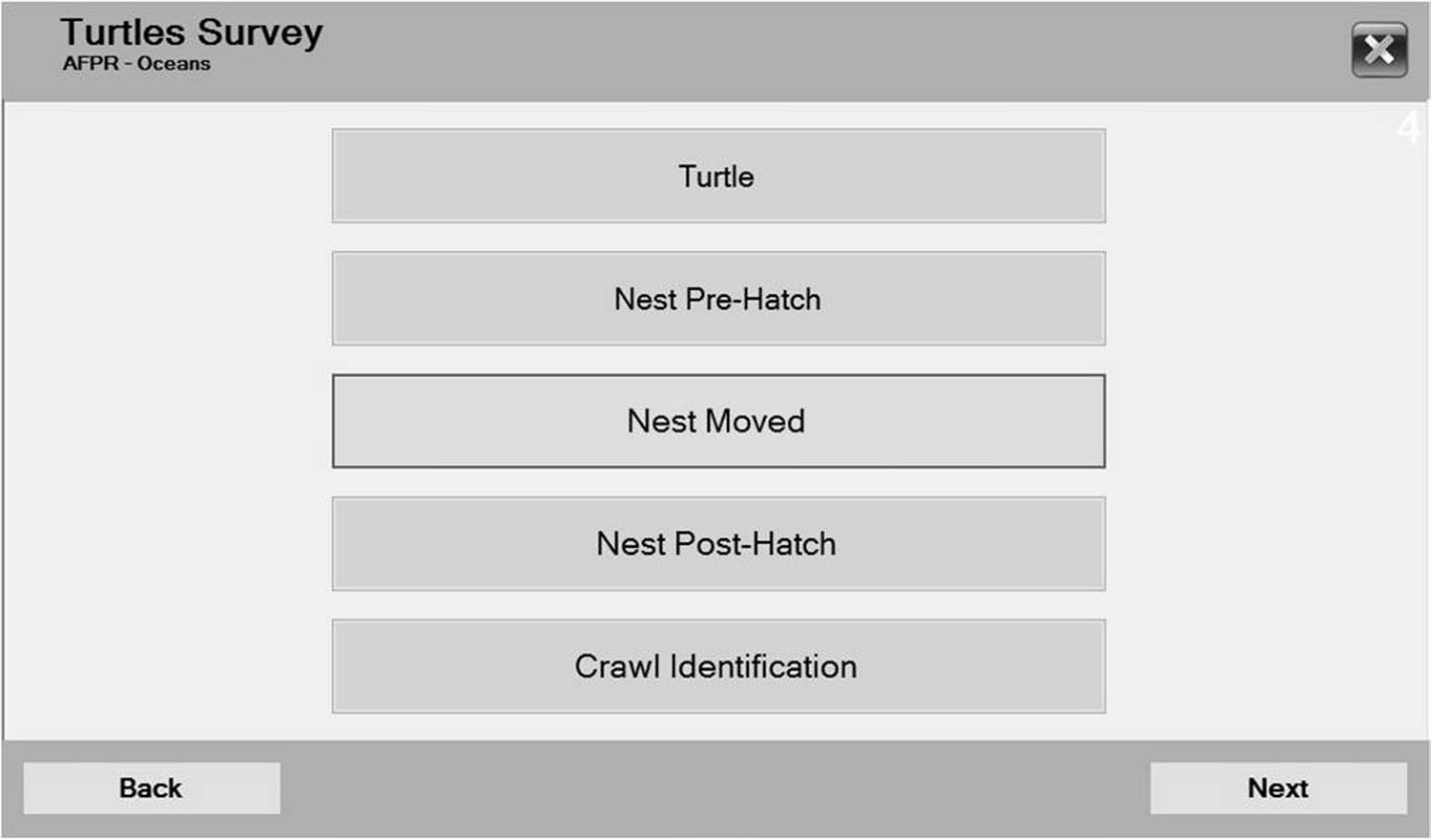
Window with menu buttons to select the data type for each case. This window enables the user to decide between boxes R1 (Turtle button), R2 (Nest Pre-Hatch button), R3 (Nest Moved button) or R4 (Crawl Identification) in the diagram in Fig. 2; each box had a set of specific variables concerning the case. A new button, “Nest Post-Hatch,” that could be a R5 box, enabled the recording of nest variables after hatching. This button and the variables concerning a nest evaluation after eggs hatched exist in the software version that was tested (see Fig. 6).

Each window has several variables. Window 4 “Select type of record” is shown in Fig. 4 and provides a menu of five buttons, since the user must decide which kind of record ought to be filled.

**Figure 5:**
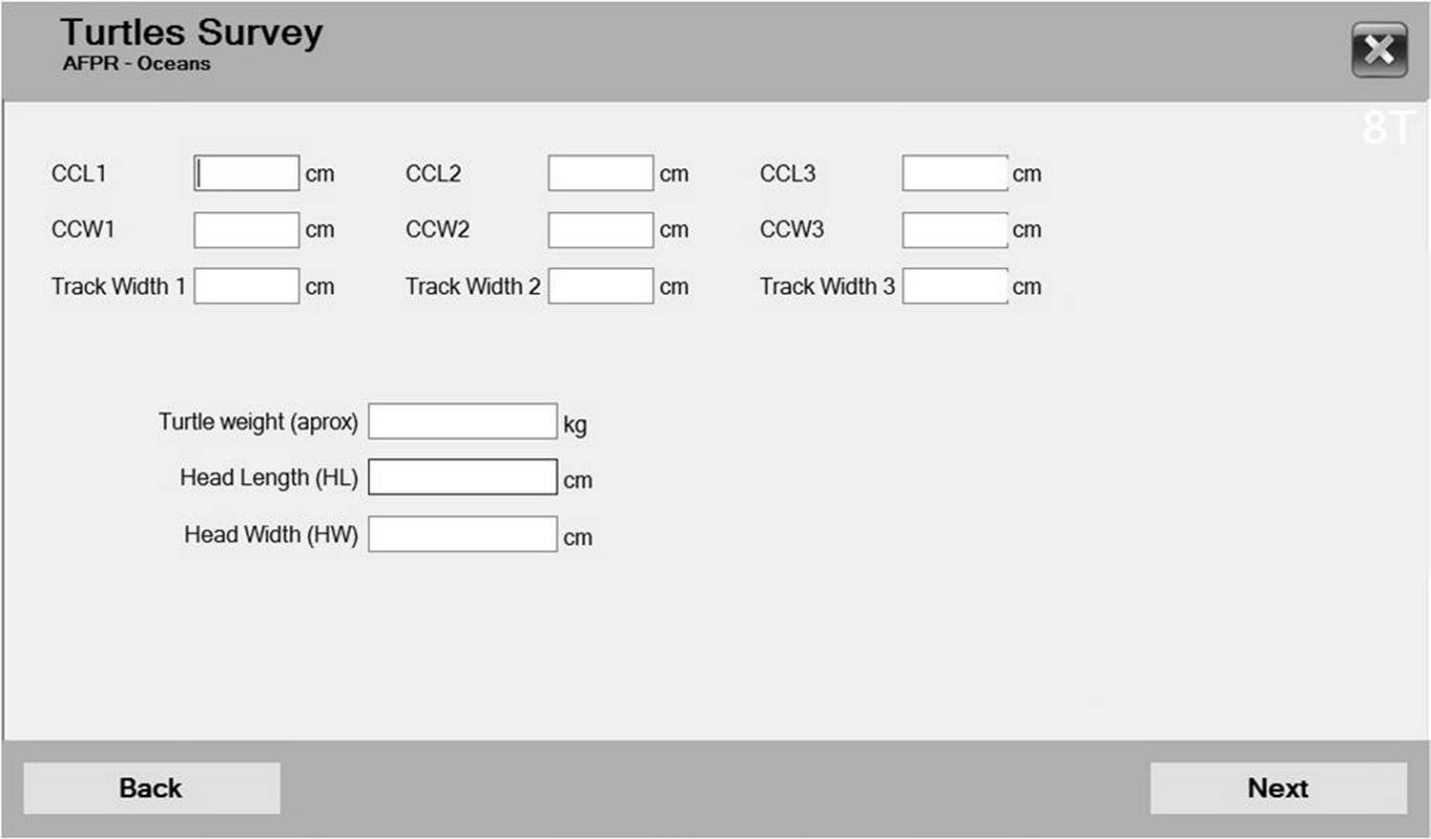
After choosing the R1 variable set (see Fig. 2), several windows appeared in sequence, such as the window shown (which corresponds to window 4.1.4. from Fig. 3). Turtle measurements, such as CCL, CCW, track width, weight, head length and head width, can be recorded in this window.

The set from 4.1.1. to 4.1.5. is about female turtles’ characteristics: 4.1.1. is the window where the user identifies the species (information for identification is provided); 4.1.2. has several fields where the user inserts information about old tags (kind, number, where it is applied), and new tags if they are applied; 4.1.3. has a diagram of a turtle’s plastron with the measurements is provided, and PTL and TTL measurements can be inserted; 4.1.4. provides a diagram of a turtle with the measurements: CCL, CCW, and track width can be inserted (for each, three fields are shown, since references^17^ advice taking each measurement three times); weight, head length and width can also be inserted (this window is shown in Fig. 5); and 4.1.5. asks for turtle activity, its health conditions, date (generated automatically), hour (generated automatically), information on additional experiments (*e.g*., codes of sample taken for DNA analysis), turtle status after leaving the beach, and distinctive marks or characteristics of the turtle and provides a field for notes (free text).

Windows 4.2.1. to 4.2.8. show a sequence of variables to record information about a nest that is found. Window 4.2.1. generates an automatic code for that nest (its tag), but the user can insert a different code after the automatic code. This code is generated with a code for the location and a number. Window 4.2.2. inserts the date (automatically). Window 4.2.3. asks for the name of the beach and area where the nest is seen (because many projects divide beaches per sections) and provides a third box to add more references. Window 4.2.4. allows the introduction of latitude and longitude of the nest and asks for type of mark that is used to identify the nest. Window 4.2.5. asks if the female is present (yes or no); if the user chooses the “yes” button, the software asks for the tag of the female. Window 4.2.6 collects information about the clutch size and automatically inserts the date of the eggs laid (redundancy); the user is asked for total number of eggs laid; if it is a partial or a complete clutch; number (#) of incubated eggs (with embryos), # yolkless eggs, and # multiyolked eggs; and time of deposition (inserted automatically but can be typed also). Window 4.2.7. is the nest data window, where the user inserts temperature of the sand (°C – measured at 50 cm of depth), sand humidity (%), location of nest along the beach (selects, or types), distance of nest to the high tide mark (selects) and inserts a value (meters); and measurements of depth of the nest (A) from the sand surface to the top of the first egg and (B) until the bottom of the egg chamber. Finally, in window 4.2.8. the user can select the kind of vegetation, disturbances and obstructions around the nest; also, the user is asked about the dune height (m).

If the software user decides to move the nest to a hatchery, windows 4.3.1. and 4.3.2. are important to record the new information; first in 4.3.1., the user selects the nest from a list of nest tags, and then the user inserts the new GPS coordinates for the nest.

**Figure 6:**
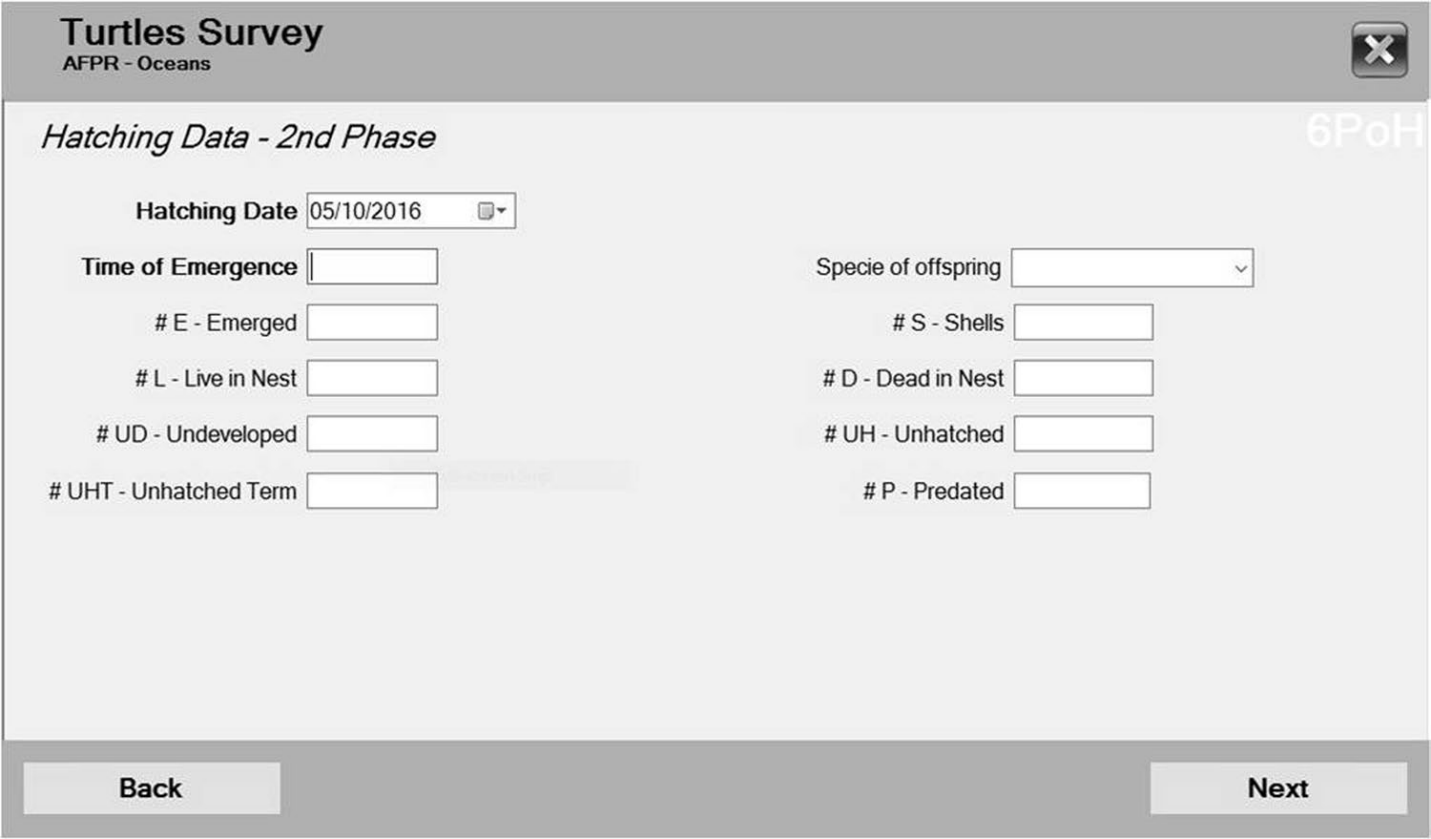
If the user had a chance to record data in box 5 (Fig. 2), the variable set to fill in after the hatchlings left the nest; the user could use this window to choose the date, type the time of emergence, and select the offspring species and the count related to the excavation categories. By filling in all the fields, the software automatically computes the emergence and hatching success rates.

After the eggs hatched, the user will add information in windows 4.4.1. to 4.4.3. Window 4.4.1. allows the user to select and recover the nest tag. Window 4.4.2. allows the user to set the hatching date (generated automatically but can be typed), time of emergence; # of emerged hatchlings; # of live hatchlings in the nest; # undeveloped; # unhatched; # shells, # dead hatchlings; and the # of predated eggs/hatchlings. The species of the hatchlings is also selected (see Fig. 6, which is a print screen of window 4.4.2.). Window 4.4.3. has fields for genetic sample codes (if necessary), and the user can choose what was the fate of the nest (flooded, invaded by predators, dislocated, or other); finally, the last field is the date of conclusion (automatically generated). If the user testimonies a “false crawl” behaviour or if the user only finds a crawl, the information can be added in window 4.5.1. choosing the type of track (buttons with images for green, loggerhead and hawksbill crawls and a button for other type); track width (cm) is asked; nest deposition (Y or N); and information about disturbance factors (from a collection of answers). If the user finds a nest, after fulfilling information in window 4.5.1., the user can skip to the “nest pre-hatch” windows.

As shown in Table 5, the fields that are pre-filled or that required one-click selection represent 41% of all fields in the software. This means that the largest portion of fields is intuitive, fast to answer and enables the reduction of potential errors in the database by narrowing the answer to a set of pre-defined options. The number of buttons and fields does not indicate the number of variables necessarily. The type-in number fields are the more numerous (40%), which is justified by the need to take measurements (carapace, depth of nests, distances to high tide watermark, etc.) and readings (of temperatures, of moisture, of tag numbers). The remaining 19% of the fields of the software are of type-in text and more prone to errors.

**Table 5:** Amount and type of fields per section and window (windows ID are the same as shown in Fig. 3).

Section	Login	Beach Weather		Record Type	Turtle Details					Nest Pre-Hatch								Nest Moved		Nest Post-Hatch			Crawl ID	Save	Total by Field Type	
**Window ID**	**1**.	**2**.	**3**.	**4**.	**4.1.1**.	**4.1.2**.	**4.1.3**.	**4.1.4**.	**4.1.5**.	**4.2.1**.	**4.2.2**.	**4.2.3**.	**4.2.4**.	**4.2.5**.	**4.2.6**.	**4.2.7**.	**4.2.8**.	**4.3.1**.	**4.3.2**.	**4.4.1**.	**4.4.2**.	**4.4.3**.	**4.5.1**.	**5**.		
Multiple Buttons	0	0	0	1	1	0	0	0	0	0	0	0	0	0	0	0	0	0	0	0	0	0	1	1	**4**	3%
Checkbox Option(s)	0	0	0	0	0	5	0	0	0	0	0	0	0	2	1	0	2	0	0	0	0	1	0	0	**11**	9%
Drop Box	0	2	6	0	0	5	2	0	3	1	0	1	0	0	0	2	0	0	0	0	1	0	2	0	**25**	22%
List Box	0	0	0	0	0	0	0	0	0	0	0	0	0	0	0	0	0	1	0	1	0	0	0	0	**2**	2%
AutoFill	0	0	0	0	0	0	0	0	2	0	1	0	0	0	1	0	0	0	0	0	1	1	0	0	**6**	5%
Type-in Number	0	3	2	0	0	1	2	12	0	1	0	1	2	0	4	5	1	0	2	0	9	0	1	0	**46**	40%
Type-in Text	2	4	1	0	0	2	0	0	3	0	0	1	1	2	0	0	1	0	0	0	0	5	0	0	**22**	19%

The version that was tested allowed the user to navigate with few restrictions. However, it is intended that the following versions have more restrictions. For example, to skip to the variables about the turtles, the user must previously fulfil data concerning weather conditions. The idea is to avoid creating databases with missing fields.

Though it is a proof of concept, the software is available by contacting the authors. The strategy is also to adapt the tool for each project focus, *i.e*., each project can set the group of variables they need in their software version.

### Pilot Study

The Turtles software was tested from June 1^st^ to July 31^st^ during the night patrols by one biologist (software user). The patrols were done between 9 p.m. and 2:30 a.m. (time varied until 3 or 4 a.m., depending on the nesting behaviour) from Monday to Friday by one biologist that accompanied one *Tortuguero*. Each night, one beach was patrolled by the pair. The two main beaches (with higher densities of emergences and nests) were patrolled alternately (Half Moon Bay and Jade Bay) by this pair during each week. Each night, the tasks were divided on the field, but all records were made by the two workers. The biologist used the software in the field, while the *Tortugueros* entered the data manually on paper forms followed by CEA staff entering the recorded data into an Excel spreadsheet (paper forms database). Comparisons were then made between exported data from the software with that provided by CEA.

### Samples and Variables for Data Analysis

The data focused on *Chelonia mydas* and *Caretta caretta*, the two species that nest in Jade Bay and Half Moon Bay (Fig. 1). After completing each record in the field (for example, per turtle, or per nest), the software allows the user to save (and add) the information to the Access table. This information is available to the user to be transferred to other programmes, such as Microsoft Excel.

From each method (traditional with paper forms and the software), samples for a variable set were chosen, since the original databases were different in organization and number of variables. Then, the two related samples (the paper forms sample and software sample) were compared without subsequent modification.

The two complete datasets were compared to assess the potential benefits and drawbacks of the software compared to the traditional methodology when building tables for statistical analysis. Information that disturbed data analysis was found (*i.e*., different texts concerning the same thing for the same variable). It implied standardization and correction of information on those variables (in the paper forms database), before statistical analysis/tests were performed. Variables of the paper form database were standardized using the Microsoft Excel 2007.

To determine whether the software methodology would interfere with the user’s performance in the field, the averages for records and data collection were determined. A field in the database is a cell fulfilled (or not, which is the concept of missing value) with information concerning a variable. A record is a complete row of fields concerning a single turtle or a single nest.

The average number of records was determined using the paper forms database as a reference to determine the performance and impact of the software in the field work. The average of fields fulfilled was also determined; the variables were gathered by categories (four categories in total, *i.e*., “Turtles seen or not seen (includes false crawls)”, “Turtles seen”, “Nests seen”, and “Nests moved”). These averages are indicators that allowed concluding if the software is suitable for field work in terms of quantity and quality of data recording. This work was done in Microsoft Excel 2007.

For the records of both databases, several data consistency parameters were analysed, such as the mismatch of content, the lack of dependent variables, and the amount of unrealistic data, which were indicative of data integrity. The analysis implied determination of the percentage of missing fields on records; GPS location of the nest; time of the record; user name and nest ID; and percentage of errors in the fields (an error implied correcting it when possible, or discarding it when the value was absurd). Errors were divided into two categories: “different answers for the same value”, and “inconsistent data”. For “different answers for the same value”, information that fitted one of the following two groups was included: “different texts for the same user” and “different texts for the same turtle false crawl reason”. The error “inconsistent data” includes the “mismatch between total number of eggs and the sum of the partials” (meaning errors in formulas that the software avoids), and “GPS data out of the range of the region” (see Table 1 in the “Results and Discussion” section).

## Conclusions

The pilot study of the Turtles software suggests it to be a more efficient and reliable method in comparison with the traditional paper forms recording methodology and the subsequent data transcription for nesting marine turtle conservation programmes. The software can help to increase the reliability of estimated trends. Moreover, it can contribute to the global standardization and sharing of recorded information. It should be made available online so it can be adopted by nesting turtle projects that are interested in replacing paper records with an easier and a more reliable solution that uses tablets or smartphones.

A software *per se* will not ensure data quality. However, if combined with a well-delineated methodology, it will certainly improve field data collection, specifically because data will be added to a database the moment the software user in the field saves the information. Additionally, software applications can use code and predefined answers, making databases intelligible. The principle that a “collective focus should be to achieve comparable, replicative results with accuracy and precision”^17^ was considered. Following this principle, an easy-to-use solution was designed to generate data that can be easily sent to statistical analysts, so that results about the collected field data can be generated but also increase the power of the field work.

### Ethics

All methods were carried out in accordance with relevant guidelines and regulations imposed by CEA (2016) and SEMARNAT (DOF 2013). Additionally, all experimental protocols were approved by the same entities.
